# Effect Model Law: An Approach for the Implementation of Personalized Medicine

**DOI:** 10.3390/jpm3030177

**Published:** 2013-08-15

**Authors:** Jean-Pierre Boissel, Riad Kahoul, Draltan Marin, François-Henri Boissel

**Affiliations:** Novadiscovery SAS, 60 Avenue Rockefeller, Lyon 69008, France; E-Mails: riad.kahoul@novadiscovery.com (R.K.); draltan.marin@novadiscovery.com (D.M.); francois.boissel@novadiscovery.com (F.-H.B.)

**Keywords:** treatment decision-making, effect model, personalized medicine

## Abstract

The effect model law states that a natural relationship exists between the frequency (observation) or the probability (prediction) of a morbid event without any treatment and the frequency or probability of the same event with a treatment. This relationship is called the effect model. It applies to a single individual, individuals within a population, or groups. In the latter case, frequencies or probabilities are averages of the group. The relationship is specific to a therapy, a disease or an event, and a period of observation. If one single disease is expressed through several distinct events, a treatment will be characterized by as many effect models. Empirical evidence, simulations with models of diseases and therapies and virtual populations, as well as theoretical derivation support the existence of the law. The effect model could be estimated through statistical fitting or mathematical modelling. It enables the prediction of the (absolute) benefit of a treatment for a given patient. It thus constitutes the theoretical basis for the design of practical tools for personalized medicine.

## 1. Introduction

**Figure 1:**
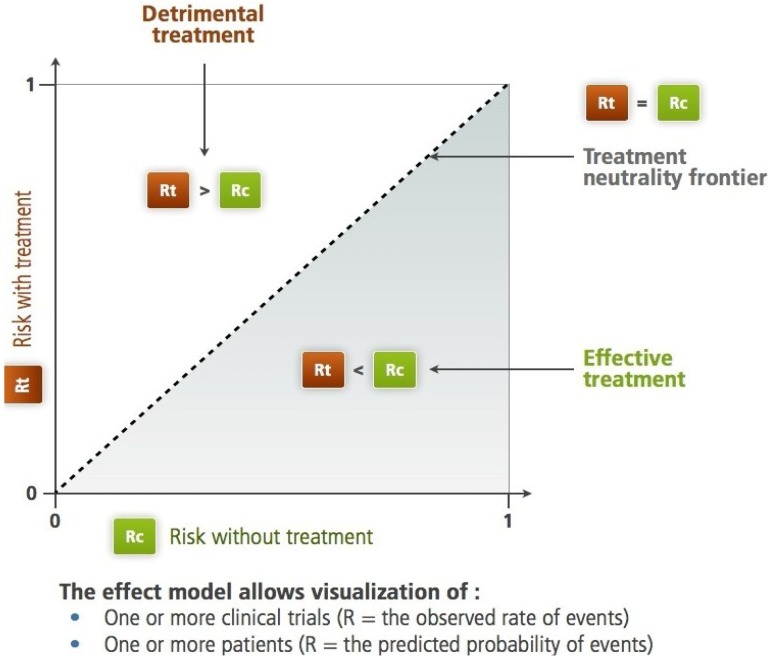
The Rt, Rc plane according to L'Abbe *et al.* For the studied criteria (therapeutic objective), the diagonal separates the harmful treatment area from the efficient treatment area. On this plane, we can identify subjects, trials, groups of subjects or populations. Two different paradigms are applicable: the frequency-based paradigm, *i.e*., what has been observed (see Figure 2) and the probabilistic paradigm, *i.e*., what the computational model predicts (prediction, see Figure 3).

**Figure 2:**
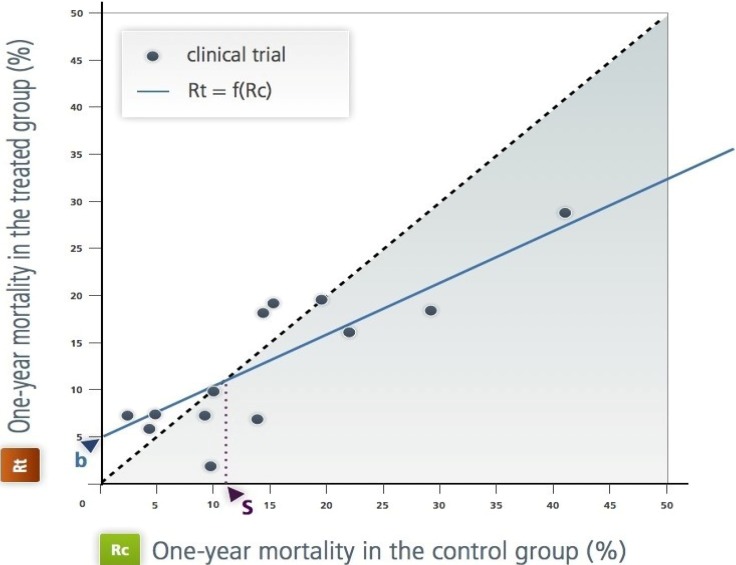
The effect model of the class 1 antiarrhythmic drugs during the year following a myocardial infarction [3]. This is as L'Abbe *et al.*’s representation in the Rt, Rc plane. Each point represents a randomized trial; the x-axis (Rc) is the frequency of the event over one year (in this case, mortality over a year) in the control group (in this case, placebo or no antiarrhythmic treatment). The y-axis is the frequency in the treated group by the antiarrhythmic drug of interest. All the published trials are represented. ‘b’ is the intercept; it is an estimation of the toxicity. ‘s’ is the ‘natural’ threshold of Rc above which the treatments have positive net efficacy. This figure illustrates the empirical approach.

In 1987, L'Abbe, Detsky and O'Rourke recommend to include a graphical representation of the various trials when designing a meta-analysis. For each trial, on the x-axis, the frequency (risk) of the studied criterion in the control group (Rc) should be represented, and on the y-axis, the risk in the treated group (Rt) [1] (Figure 1, Figure 2).

The shape of the resulting scatter plot illustrates some important aspects of the information concerning the effect of the treatment.

In 1989, Lubsen and Tijssen used this kind of representation and suggested that a treatment with “an average” benefit may be harmful in patients at low risk [2]. However, they did not base their proposal on the analysis of real data.

Without prior knowledge of Lubsen and Tijssen’s article [2], Boissel *et al.*, while studying the effectiveness of antiarrhythmic drugs in the prevention of death after myocardial infarction by using the meta-analysis approach, noted that regardless of the metric chosen to measure the “average” observed efficiency, the heterogeneity between results of trials persists, which is inconsistent with standard statistical assumptions of meta-analyses. They showed that this can be explained by focusing on the relationship between Rt and Rc of these antiarrhythmic drugs, a relationship they called “effect model” in an article published in 1993 [3] (Figure 2). For these drugs, the relationship seems peculiar, with the presence of an Rc threshold below which they induce more deaths than they prevent. This illustrates the intuition that all doctors have, and that which Kaurer and Kassirer emphasized in 1980: a treatment can yield little benefit; even worse, it can be more harmful than beneficial for “moderately sick” patients [4]. The approach followed by Boissel *et al*. is based on a model that combines a beneficial effect that is proportional to Rc and a constant adverse effect, independent of Rc. The mathematical expression of this model is a linear equation with two parameters, the risk of lethal adverse event caused by treatment and the slope of the line which represents the true beneficial risk reduction:

*Rt = a. Rc + b* (1)

where *a* carries the beneficial effect and *b* carries the constant lethal adverse effect.

**Figure 3:**
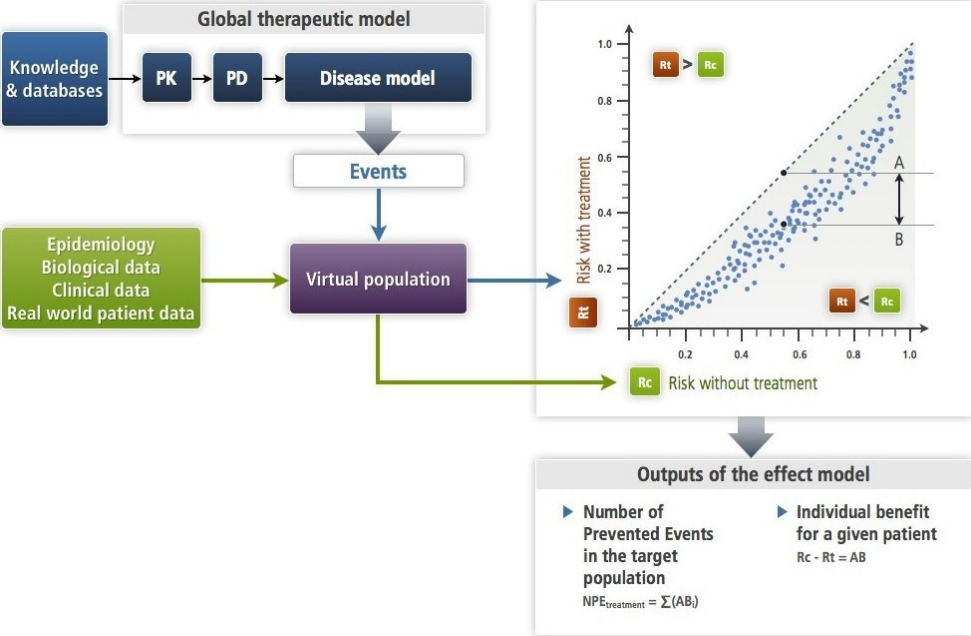
This figure illustrates the simulation approach (Section 2.2) with a mechanistic disease model (Section 4.2) as it has been formalized [6]. The input is an amount of drug—single or repeated dosing—the PK-PD brings to its site of action where its target is altered as a function of its concentration. This target bridges with the disease model (see Section 4.2). The PK-PD and the disease models make up the therapeutic model. Each subject of the virtual population is submitted in turns to the disease model alone (Rc) and to the therapeutic model (Rt). This results in an estimate of the effect model for this drug, the disease, the event and this subject. One can compute the absolute benefit for each patient, while summing absolute benefits across all patients gives the number of prevented events. In this approach, the models (disease and therapeutic) are deterministic. Note that the virtual population enables to account for the known or presumed biological variability.

This equation gives the treatment net mortality reduction. By fitting this equation to the available data through a statistical regression technique, the authors estimated the parameter values and inferred the value of the threshold. In theory, only patients whose risk without treatment is above this threshold should be treated (Figure 3 and Section 4.1). The same model will be used later for aspirin [5].

Building on these previous ideas and introducing their own personal experience, in 1995 Glasziou and Irwig analysed the relationship between Rt, Rc built upon results of randomized clinical trials [7]. 

In 1998, in an editorial accompanying the publication of a study about the risk of bleeding with aspirin therapy, Boissel applied what would become the effect model law to this medication used in cardiovascular prevention. He showed that for subjects with low risk of cardiovascular events, aspirin is probably harmful [5].

At the end of the 90s, a set of studies dealing with the generalization of this relationship was published [5,8,9]. Among other developments, they suggested that Rc should be replaced by a set of patient descriptors as an independent variable. However, for historical and practical reasons, we prefer to keep the ‘Rt, Rc relationship’ wording. Their results, presented in Section 2, led to the notion of a “law” of the effect model. Little was heard over the next decade about the Rt, Rc relationship.

The relation between Rt and Rc is defined for a triplet Disease, Event, Treatment (DET) with *t* the duration of follow-up. The law can be phrased as follows:

“For a given treatment, a given disease and a group of subjects, there is a relationship between the course of the disease in untreated subjects and the course of disease in the same treated subjects. This relationship is usually represented in the Rt, Rc plane where Rc and Rt are the rate or probability of the disease outcome in, respectively, untreated and treated subjects. The relationship can be empirically approached—at least in some cases—by a function. This functional representation is important for it enables the computation of the rate or the prediction of the probability of the disease outcome (Rt) in the treated subjects and their absolute benefit. In other instances, Rt and the absolute benefit can be derived by simulation.”

## 2. Substantiations of the Reality of the Law

### 2.1. Empirical Approach

The first empirical case was the study of antiarrhythmic drugs in post-myocardial infarction. The effect model was built from published results of randomized clinical trials (summarized data, see Section 1, Section 4.1, and Figure 3). The frequency of death in the control group provided the values of Rc and the frequency of death in the treated group with antiarrhythmics provided Rt. Other cases were then studied, essentially in the cardiovascular field (e.g., antihypertensive therapy, cardiovascular prevention with ACE inhibitors or statins, *etc*.). In these studies, the individual data of the trials allowed the verification of the existence of the relation, and to expand the estimation techniques [9].

### 2.2. Approach by Simulation

It consists in modelling mathematically the disease and the treatment, in generating virtual individuals (virtual population: realistic or not) and in applying the disease model and then the therapeutic model (treatment acting on the disease) to every virtual subject [10]. The outcome is always a connection between Rt and Rc, which is represented in the Rt,Rc plane (L'Abbe *et al*’s graph). This relationship can be linear or curvilinear, with a total of five different situations (see Figure 1 in [11]. The advantages of this approach by simulation make it invaluable. First, it enables the exploration of this relationship for every possible risk value of the studied event. It also allows for the structured exploration of the values of patient descriptors that play a role in the relationship. Another advantage is the precision of the prediction, which depends only on the computing time and adequacy of the model to the knowledge used to build it (controllable factors) and the uncertainty of this knowledge (non-controllable, but open to estimation). We emphasize that in this approach, the Rt, Rc relationship is not introduced explicitly in the modelling process. Rather, it is a result of simulations, and therefore is intrinsic to the simulated phenomena, disease, treatment, and patients. Finally, this approach allows the specification of the respective roles of two types of patient descriptors (X and Y, see Section 3) implicated in the process.

In order to illustrate this approach, let us take a sham case that Boissel *et al.* utilized to explore the attributes, features and consequences of the Rt, Rc relationship [10]. The drug was given at dose D. They then followed six steps—absorption, distribution, drug-receptor interaction, post-drug-receptor transduction, homeostatic reaction and clinical transduction—through which the drug was carried to its target where its effect appeared. Interaction with its target resulted in a change in the probability of the simulated clinical event. Each step was described by one or more algebraic equations derived from classical pharmacokinetics and pharmacodynamics modelling. A one-compartment distribution was assumed. The probability of event was given by a logistic function. Equations are shown in Annex A. The whole model includes six variables or patients descriptors linked with the pharmacokinetic and pharmacodynamics interactions of the drug with the body. These are the X variables: a composite variable that carries subject-specific molecule absorption factors, age, a variable that summarizes expressions of genes that determine the maximum stimulus generated when the drug binds to its target, another variable that carries the expression of genes that modulate drug-target binding, a composite variable combining the effect of various allele expressions, ionic strengths, signal transducer availabilities that modulate the signal emerging from target alteration, and a variable called sympathetic drive that modulates the feed-back on the strength of the pharmacological effect. Eventually, there were two Y variables (patient descriptors) specific of the disease: (i) a biological variable that is key in the disease mechanism; its relationship with the risk of the clinical event is modified by the drug through the final alteration of the target; (ii) a risk factor that is not altered by the drug. The eight variables (X + Y) were used as descriptors for the virtual population. Distributions were assumed to be either Normal or log-Normal. Several population sizes were used. Plot in Figure 3 has been obtained with size 200.

### 2.3. Theoretical Approach

Using fundamentals of pharmacology (Hill’s model) and an extremely simplified representation of the probability of a morbid event (logistic function), Wang *et al.* [12] tried and succeeded in reproducing this relationship. Mathematical derivation is shown in Annex B. The resulting equation between Rt and Rc is:

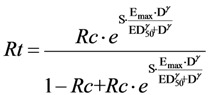



Note that in this simple theoretical setting, the Rt, Rc relationship is curvilinear. 

This is another confirmation of its existence because, as with the simulation approach, the relationship is not explicitly introduced into the equations expressing the phenomena.

## 3. Major Features of the Relationship

### 3.1. Population-Level and Individual-Level

An empirical context is ill-suited to explore individual effect models. Evidence for individual effect models for a given triplet DET arises from the mechanistic approach. When the global therapeutic model (see Figure 3) has been validated and the virtual population has been designed, drawing a single virtual subject and replacing in the model the variables by the patient’s corresponding descriptor values results in an *individual* effect model. The relation between Rt and Rc, or better, the function that gives the value of the absolute benefit with a treatment T at a time *t* is specific to the values of X and Y. This enables to account for the “within patient” variability when necessary.

### 3.2. Patient Components of the Effect Model

Patient components are more accessible when the simulation approach is used. Two categories of patient components, which are represented by as many patient descriptors, have been identified as determinant of the shape and the situation of the relationship for a group or a patient: some patient characteristics are linked to the disease (genotypic, phenotypic, environmental), and determine the value of Rc. Others express the interactions between the patient and the treatment (for example, for a drug, the determinants of the volume distribution or of the absorption speed). These descriptors determine the y-axis value according to the average Rt value in the population for the given Rc. Some patient descriptors can be common to the two categories. The values of the first category will be called Y and the second X (see Figure 2 in [11]). Another element is involved: the iatrogenic effects, which are expressed through the same event as the one the treatment is supposed to prevent (for example death). The case of the antiarrhythmic drugs previously mentioned illustrates this situation. Lubsen and Tijssen had predicted this. The distributions of all these descriptors enable us to design the virtual population (see Figure 3).

### 3.3. Expressions of the Law

The relation between Rt and Rc is defined for a triplet Disease, Event, Treatment (DET) with *t* the duration of follow-up. Treatment may represent a particular drug, as in the example of a drug preventing angina pectoris attack in Section 4.2 or a class of drugs, as in the example in Section 1 (and Figure 2). 

In order to derive operational tools, the effect model law can be expressed symbolically in two ways.

(1) the Rt function:
  *Rt = f(Rc,X,T,t)* or *Rt = g(Y,X,T,t),* equation in which Rc is implicit;
(2) the absolute benefit function, AB:
  *AB = Rc-Rt = h(Y,X,T,t),* equation in which Rt and Rc are implicit.

The symbolic forms above put forward the variables that are behind the relation: the two types of patient descriptors X and Y, the treatment of interest and time. These forms show that there are as many values in the Rt, Rc plane as there are patients, each one being represented by a dot which is more or less close to the average curve. The expression of the absolute benefit AB has the advantage of leading directly to an individual prediction.

It should be stressed that, except for the statistical approach (as with the antiarrhythmic case in Section 1) and a few simple cases of phenomenological disease modelling (as shown in the theoretical approach in Section 2.3), there is not a unique and global mathematical equation substantiating either one of these symbolic forms.

### 3.4. Basis for the Effect Model Law Consequences

Consequences of the law that lead to applications in personalized medicine are based on quite a simple derivation from its absolute benefit function expression: *AB = h(Y,X,T,t)*.

The absolute benefit is the best expression of what a patient can expect from a treatment because this index tells what the patient would gain in terms of morbi-mortality or quality of life by being treated with T. Other indices such as the relative risk or the odds ratio do not carry the same information. Thus, it is the sensible benchmarker for individual decision making in choosing between T_i_ and T_j_. Further, as explained later in this article, the prediction of the absolute benefit could be compared to a threshold, whether it is community or individually-based.

When summing all predicted ABs of patients in a group or in a population, one computes the number of patients who would have suffered an event had they not been treated, or the number of prevented events for an outcome that cannot recur. This is shown in Figure 3.

### 3.5. Representation of Effect Models

Considering the average value of Rt for each Rc, there are five possible representations (Figure 1 in [11]):

A straight line crossing the x- and y-axes at 0;
A straight line with a threshold crossing the y-axis at Rt >0;
Curvilinear;
Curvilinear with lower and/or upper thresholds.

### 3.6. Role of Time

The Rt, Rc relationship for a treatment is specific of a therapeutic objective and a set of patients. It also depends on the duration of observation. That is why an “instant” form of the symbolic expressions may be preferred, in the same way we talk about instant risks and hazard ratios. It is even more relevant when it appears that the therapeutic benefit for a chronic disease is not necessarily constant [13,14]. However, taking time into account raises major difficulties, mainly the lack of available data with the statistical approach. With the simulations approach, it can be feasible at the price of important computational time. That is why it is usually more convenient to set the duration of observation.

### 3.7. Two Paradigms

The effect model law, the Rt, Rc relationship and its representation in the Rt, Rc plane can be considered according to two distinct perspectives: the Rt, Rc frequencies and the Rt, Rc probabilities. The first comes from the statistical paradigm: we are querying backward-looking data. To do so, we rely on data collected during clinical trials. The second is forward-looking. We are here in the prediction paradigm, with the caution such an approach commends. However, there is of course a link between the two perspectives. Predictions rely on the past, *i.e*., the knowledge generated by researchers but also on data (used to calibrate some of the model’s parameters and to validate these models, with an independent dataset in the latter case).

## 4. Estimation Methods and Prediction of the Relationship

There are two approaches to estimate the true effect model, which are quite different in terms of data needed, modelling and generalizability of the outcomes. 

### 4.1. Statistical Approach

Classic statistical regression approaches apply when working with data from clinical trials, either summarized or individual data. For instance, in the antiarrhythmic case (Section 1, Section 2), fitting of Equation (1) to available clinical trial summarized data gave the estimates of *a* and *b* for one year of treatment duration [3].

*a* = 0.56 ± 0.18
*b* = 5.3 ± 2.6 in (%)

Sensitivity analysis did not change these estimates in a material way (e.g., *a* from 0.52–0.62). Other polynomial models did not fit as well. These values were used to design the straight line on Figure 2. Effect model or derivatives such as the absolute benefit are highly dependent on the data their estimate is based on. Validation with new data is important. However, it does not guarantee generalizability, which is certainly a hurdle when the objective is personalized medicine.

### 4.2. Mechanistic or Phenomenological Modelling Approaches

These approaches do not rely on clinical trials data, except for the calibration of some model parameters and the validation of these models. They are based on a thorough analysis of available knowledge about the disease and the therapy, which is then processed into formal models (series of mathematical solutions: algebraic equations partial differential equations, partial derivative equations or others, such as multi-agent solutions, or combinations). To be functional, these models are combined with virtual populations, whether realistic or not [10,15,16]. In most of these situations, especially when the number of, e.g., differential equations is large or with a multi-agent approach, it is not possible to represent the whole model by a single equation. In such a case, if the objective is to predict an absolute benefit for a given patient or a number of prevented events (see Figure 3) for a given population, the simulation approach has to be used.

Several examples of this approach have been published [10,15,16]. One is summarized here and in Annex C [17]. The aim of this work was to predict the beneficial effect of a new chemical entity in the prevention of angina pectoris attack (AP). Wet lab information and data from a phase I dose-ranging study on healthy volunteers were utilized to design the PK-PD sub-model (see Figure 3). The disease model was based on available knowledge on the mechanism of AP. A series of nine algebraic equations described the mechanism leading to the onset of an AP (see Annex C). The virtual population has been constructed with real data drawn from a cohort of 1,706 real normal subjects with 24-h heart rate and blood pressure recording. Onset and time of onset of AP for each subject of the virtual population was computed by applying the disease model to each subject and recorded. Then, the drug was given at various doses to each subject through the application of the therapeutic model and the onset of AP was computed and recorded. Comparing individual occurrence of AP with (Rt) and without treatment (Rc) and summing across the 1,706 virtual subjects yield the effect model (see Figure 4 in [10]) and the number of prevented events compared to placebo for each dose. Drawing random samples from the virtual population allows the computation of prediction intervals for the predicted outcomes (again see Figure 4 in [10]).

### 4.3. Assumptions and Caveats

With the statistical approach, assumptions are needed (e.g., normal distribution). In addition, one should be cautious when estimating the effect model by fitting regression lines on summarized clinical trial data (as in reference [3]) instead of individual data. Sharp *et al.* and others showed that the estimate is flawed because of the regression to mean phenomenon. Estimates are reliable only under certain conditions [18].

With the mathematical modelling approach, the two main potential pitfalls stem from the integration of knowledge and its mathematical representation. The former risk arises when insufficient knowledge is accounted for. The main problem here is that knowledge of disease mechanisms is usually incomplete. A particular type of knowledge frequently missing, or at least often imprecise, is the variable distributions and parameter values. The modeller makes assumptions, the strength of which should be carefully reviewed and tested. The latter risk stems from inappropriate mathematical solutions being used. 

Altogether, these limitations impose a rigorous approach to model validation. For instance, data and/or knowledge utilized to test the model should not have been used to design the model.

### 4.4. Estimating the Effect Model: From Theory to Practice

Since mathematical form, nature and number of parameters and variables depend on the disease (D), treatment (T), and clinical event of interest (E), there is no general mathematical expression for *Rt = g(Y,X,T)* and *AB = Rc − Rt= h(Y,X,T)*. Further, the form of the estimated relationship depends on the techniques which have been utilized. For the same triplet DET, statistical fitting to clinical trial data and mechanistic modelling cannot result in the same estimate of the true relationship. The latter is assumed to be more flexible, e.g., enabling the structure of the estimated model to be closer to the real one, and more precise since it can account for variables that are known to exist but were not measured in the available clinical trials. 

## 5. A Few Consequences

We will limit here the overview to the consequences in terms of public health assessment and treatment decision-making. It should be emphasized that there is a number of applications ranging from discovery (e.g., new target identification [19]) to clinical development (e.g., clinical trials design [17]), which fall outside of the scope of this paper. Additional information may be found on Novadiscovery’s website [6]. 

### 5.1. Misleading Use of Efficiency Indices

According to the effect model law, the individual absolute benefit of a therapy varies from one patient to another. Also, the number of prevented events, all other things being equal, varies from one group of patients to another, and from one population to another. These values are determined by the distributions of X and Y in these sets. Therefore, the number of subjects to treat, which is the inverted function of the absolute benefit, varies in the same way. If its value was inferred from the result of a clinical trial or a meta-analysis, it could not be considered characteristic of a therapy [20].

### 5.2. Introduction of a Threshold

As shown in the example of antiarrhythmic drugs, a therapy can be beneficial for some patients and increase the risk for others. In addition to these thresholds, called “natural” because they are implicit in the studied phenomenon (the triplet: disease, clinical event, therapy), we can apply constraints that are external to the system; for example, a risk, whether constant or not, of side effects that does not express through the same event than what the therapy is supposed to avoid, or the amount allocated to the reimbursement of prescriptions [11,16]. In all cases, the external constraint leads to a threshold below which the cost or inconveniences exceed the expected benefit. This notion of implicit threshold in the effect model law is legitimate in other ways too: it has been proposed on the basis of logical reasoning [4] and some doctors put it into practice, albeit in an informal way [21].

In reference [16], an example of a community-based threshold is presented where the external constraint is the resources a private or public health insurance system allocates to treating a particular disease (prevention of cardio-vascular events with satins). 

Regarding individually-tailored thresholds, an application will need further practical developments, such as scoring the side effect impairment of patient wellbeing and quality of life with the same scale to the outcome of interest.

### 5.3. Prediction of Individual Absolute Benefit: Towards Personalized Medicine

The second expression of the effect model law enables the prediction of the individual benefit (AB). If several treatments are competing to achieve the same therapeutic objective, the different predicted ABs can be compared for a given patient (same X, Y and different functions). So, in theory, this formulation of the effect model law allows us to personalize treatment decisions. It has been shown, first by a logical demonstration and then by simulation in a real case, that an approach of individual therapeutic decision-making based on the effect model law would usually be more efficient and more ethical (for individuals as well as from a collective point of view) than a decision-making approach based on clinical practice guidelines [11,16]. The form of the AB function depends on the form of the effect model: a linear effect model corresponds to a linear AB function where the AB increases with Rc. A curvilinear effect model corresponds to an AB function with a maximum and a progressive reduction of AB for higher values of Rc.

For a given triplet DET, the effect model is specific (by construction) to each competitive treatment. Actually, although the disease model is the same, the therapeutic model varies with drugs since the pharmacokinetics and pharmacodynamics and often the target in the disease model change with each drug. Thus, one can compare for a given patient defined by Y and X the absolute benefits expected with the available competitive treatments.

### 5.4. Prediction of the Public Health Impact

Summing up the individual benefits (AB) across the virtual population of patients yields the number of prevented events (NPE) for a fixed period of time. This is a measure of the public health impact when the virtual population is realistic, *i.e*., constructed with real world patient data. It should be noted that if the procedure is applied to patients included in a cohort, we could obtain the number of prevented events within the cohort due to therapy of interest, which no other method can achieve. It is possible to validate, at least partially, this prediction. In its recent recommendation in the relative assessment of pharmaceuticals published in February 2013, the European Network for Health technology Assessment (EUnetHTA) promotes this approach [22].

Along similar lines, the effect model law opens up avenues for the exploration of the heterogeneity of treatment effects (HTE), over and above traditional methods reviewed in the document published by the PCORI (Patent-Centered Outcome Research Initiative) initiative [23].

## 6. Conclusions

The effect model law says that there is a relationship between the risk of event (or, depending on the context, rate of event, size of a continuous outcome such quality of life, concentration of a chemical, *etc*.) without or with treatment in an individual and in a group. It does not say that the relation is intrinsically mathematical in nature. The effect model law belongs to a number of laws that have been formulated to summarize constant behaviours in living organisms and systems, such as, for instance, Darwin’s five evolution laws. However, we do not know how to represent the consequences of the effect model law in an operational way without using mathematical solutions (or statistical or numerical, e.g., multi-agent, in some instances). 

For a given subject, from the knowledge of the effect model and the valuation of the X and Y descriptors, for every available therapeutic, the estimation or prediction of the absolute benefit enables the selection of the most efficient treatment for this patient. The individualization of the threshold, by taking into account predictable adverse effects for each one of these therapeutics, for every subject, should enable the individualization of the prediction of the benefit/risk ratio. In pharmaco-economic terms, the determination of the threshold, taking into account the amount of spending that the community has decided to devote to a treatment or a particular disease’s prevention, effectively yields efficiency in the medical decision. The necessary algorithm can be installed on the practitioner’s computer and can be connected to the electronic health record for the valuation of the X and Y descriptors.
